# Needle-Free Immunization with Chitosan-Based Systems

**DOI:** 10.3390/ijms19113639

**Published:** 2018-11-19

**Authors:** Bijay Singh, Sushila Maharjan, Princy Sindurakar, Ki-Hyun Cho, Yun-Jaie Choi, Chong-Su Cho

**Affiliations:** 1 School of Engineering and Applied Sciences, Harvard University, Cambridge, MA 02138, USA; bijaysingh@seas.harvard.edu; 2 Research Institute for Bioscience and Biotechnology, Kathmandu 44600, Nepal; smaharjan@bwh.harvard.edu; 3 Division of Engineering in Medicine, Brigham and Women’s Hospital, Harvard Medical School, Cambridge, MA 02139, USA; 4 Department of Biology, College of the Holy Cross, Worcester, MA 01610, USA; psindu20@g.holycross.edu; 5 Department of Plastic Surgery, Cleveland Clinic, Cleveland, OH 44195, USA; chok@ccf.org; 6 Department of Agricultural Biotechnology and Research Institute for Agriculture and Life Sciences, Seoul National University, Seoul 08826, Korea

**Keywords:** chitosan, mucosal vaccine, needle-free immunization

## Abstract

Despite successful use, needle-based immunizations have several issues such as the risk of injuries and infections from the reuse of needles and syringes and the low patient compliance due to pain and fear of needles during immunization. In contrast, needle-free immunizations have several advantages including ease of administration, high level of patient compliance and the possibility of mass vaccination. Thus, there is an increasing interest on developing effective needle-free immunizations via cutaneous and mucosal approaches. Here, we discuss several methods of needle-free immunizations and provide insights into promising use of chitosan systems for successful immunization.

## 1. Introduction

Needle-based immunizations are the most effective and common method of vaccine administration. Despite their successful use, needle-based immunizations have some limitations. The important issues are the reuse of needles and needle stick injuries that cause many infections in patients as well as healthcare workers. Another problem is the low patient compliance due to pain and fear of needles during immunization. Besides, needle-based immunizations are not apt for mass vaccinations during emergencies such as an outbreak of pandemic diseases or infectious agents. Hence, needle-free vaccines have garnered much attention due to their ease of administration, high level of patient compliance and the possibility of mass vaccination. As a typical example, the oral polio vaccine has established itself as a successful model of needle-free immunization. Following the marked success of the polio vaccine over five decades ago, many other needle-free vaccines were conceived but only a few of them could be commercialized. Among them, oral rotavirus, oral cholera, oral typhoid fever and nasal influenza are available in the market today.

**Figure 1:**
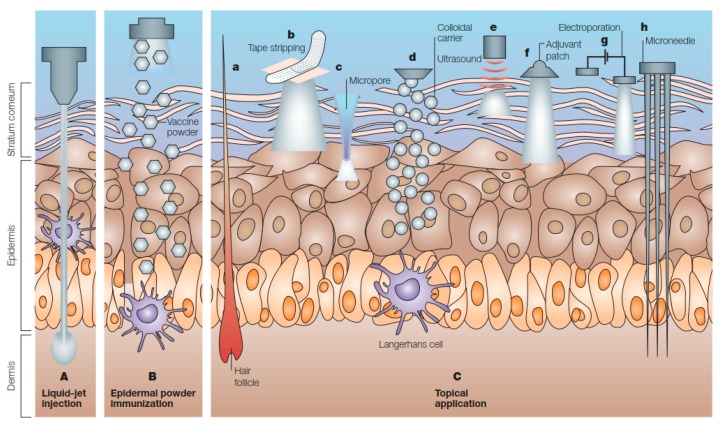
Immunization by cutaneous routes. A. Liquid-jet injection delivers vaccine to muscular, subcutaneous or dermal regions, depending on the parameters of the injection, B. Epidermal powder immunization delivers vaccine powders to the superficial layers of the skin, C. Topical application of vaccines delivers vaccines to the epidermis, where they are recognized and processed by Langerhans cells. Immunization by topical vaccine application is facilitated by several methods (a–h) (Reprinted with permission from Ref. [1]).

Needle-free immunization can be broadly classified into two major classes, cutaneous immunization and mucosal immunization, depending on the routes of vaccine administration. Cutaneous immunizations deliver the vaccines through intradermal, subcutaneous or intramuscular routes (Figure 1). Following the inoculation of a vaccine through cutaneous route, the vaccine antigens produce a local innate immune response which summons antigen presenting cells (APCs) to capture the antigens. These APCs then migrate to local draining lymph nodes to present the captured antigens to B cells and/or T cells to generate effective immune responses. Hence, subcutaneous or intramuscular routes are commonly used for vaccine administration because they are comparatively easy to access, facilitate consistent delivery and provide efficient immunogenicity among individuals. However, subcutaneous or intramuscular injection of vaccines is less effective at generating mucosal immunity compared to mucosal vaccination.

**Figure 2:**
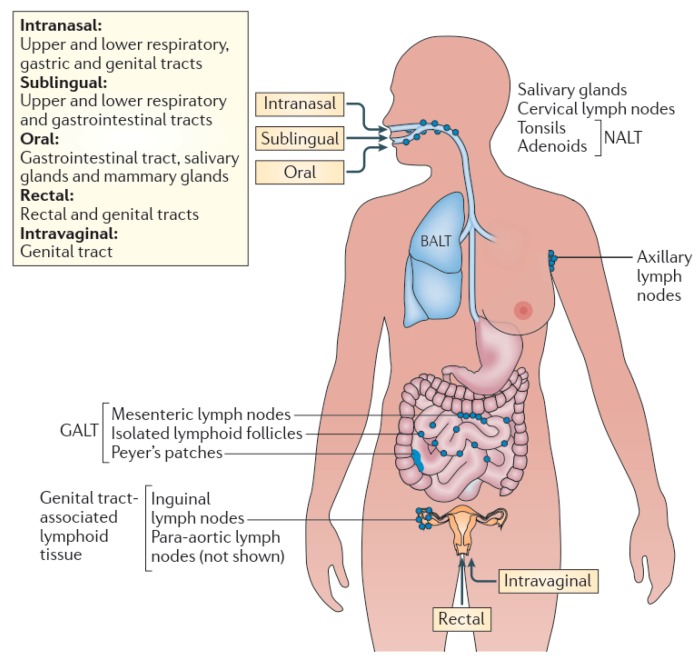
Mucosal immunization routes and compartmentalization of effector functions. Within the mucosa-associated lymphoid tissue (MALT), sub compartments can be identified, such as the nasopharynx-associated lymphoid tissue (NALT), bronchus-associated lymphoid tissue (BALT), gut-associated lymphoid tissue (GALT) and genital tract-associated lymphoid tissue. Certain immunization routes are more effective at stimulating immunity within specific, most often closely located, sub compartments of the MALT. Intranasal vaccination is preferred for targeting the respiratory, gastric and genital tracts; oral vaccination is effective for immunity in the gut and for the induction of mammary gland antibodies (which are secreted in milk); rectal immunization is best for the induction of colon and rectal immunity and to some extent genital tract immunity; and intravaginal vaccination is the most effective for antibody and T cell immunity in the genital tract. (Reprinted with permission from Ref. [3]).

**Figure 3:**
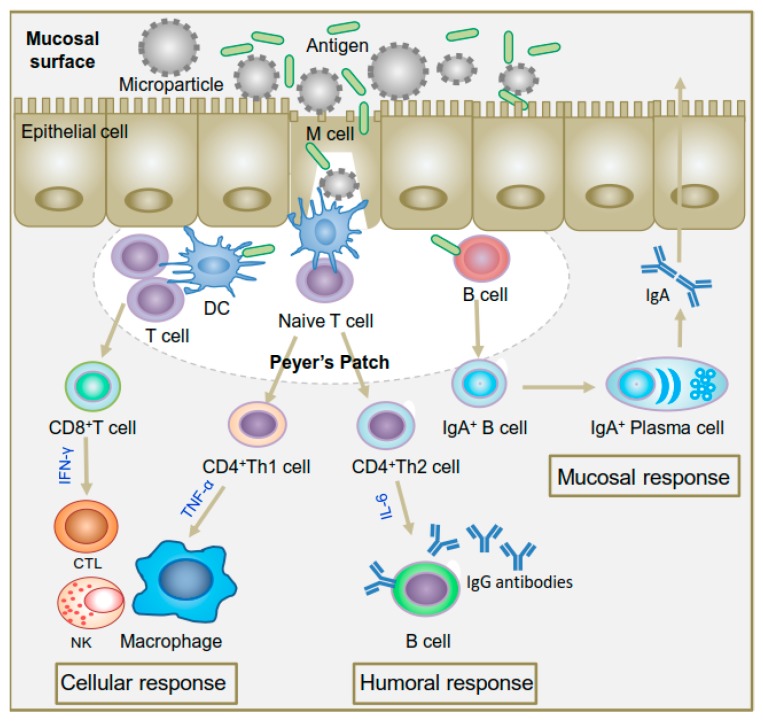
Schematic diagram of various immune responses induced by particulate vaccine system. Upon encounter with an antigen, B cells convert themselves to antibody secreting plasma cells that produce antibodies for excreting the pathogens to mucosal surfaces (mucosal response) whereas dendritic cells (DCs) present the antigen via major histocompatibility complex (MHC) class I and class II molecules to CD8+ and CD4+ T-cells. Activation pathway of CD8+ T cells and CD4+ Th1 cells produces cytotoxic T lymphocytes (CTL) and activated macrophages that kill intracellular pathogens or infected cells (cellular response) while activation pathway of CD4+ Th2 cells produces activated B lymphocytes that secrete antibodies for neutralization of extracellular pathogens (humoral response) (Reprinted with permission from Ref. [6]).

Unlike cutaneous immunization, mucosal immunization is administered through mucosal routes (Figure 2), the same routes which many pathogens harness to enter into a host for infection. Once the pathogens or vaccines enter in the mucosal routes, the local mucosal immune system generates an array of immune responses against the invading pathogens to protect the body (Figure 3). The mucosal vaccines are capable of inducing humoral, cellular and mucosal immunity [2,3]. While several mucosal routes (ocular, nasal, pulmonary, oral, vaginal and rectal) are accessible for vaccine administration, the oral and nasal routes are more appealing because they can effectively induce immunity not only at the local site of vaccine administration but also at the distant mucosal sites resulting in systemic responses [4,5]. Although vaginal and rectal routes of vaccine administration may generate effective immunity, the routes are less acceptable due to social, cultural and practical reasons.

Also, there are several other challenges for mucosal immunization. Because different mucosal routes are anatomically varied, the first challenge in mucosal immunization is determining an optimal target tissue. For example, oral and nasal immunization strategies have two discrete target areas where immunization via the oral route meets intestinal tract while nasal immunization targets respiratory tract. Scientific methods to evaluate the mechanism of immune responses from different target tissues are not yet fully understood to develop appropriate mucosal vaccines. Another challenge for mucosal immunization is ensuring the delivery of an effective dose to the target tissue which is critical to generating appropriate immune reactions. However, antigens delivered through oral route are degraded by proteolytic enzymes and acidic environment of the gastrointestinal tract [7] while antigens delivered through nasal route are readily cleared from the nasal cavity [8]. Because the anatomy and physiology vary across individuals, the accurate dose will also be varied in each person. On the other hand, safety concerns represent further challenges for mucosal immunization. Mucosal delivery of live virus or bacterial vaccines might pose a high risk to individuals with a weakened immune system. Another major challenge of mucosal vaccine development is the lack of reliable correlates of protection for mucosal immunity that would predict vaccine efficacy. In contrast, several assays are available for systemic immunity with well-defined criteria and true correlates of protection against many infectious diseases. Hence, the absence of accepted correlates of protection induced by mucosal vaccines hinders the development of safe and effective mucosal vaccines for humans.

With so many challenges, new delivery technologies to address the challenges of mucosal immunization are required. Among several delivery systems, polymeric particles as vaccine delivery systems have garnered much attention due to various advantages. For example, polymeric particles with antigens mimic natural pathogens and hence readily captured by APCs for subsequent immune reactions [9]. Another advantage of polymeric carriers is the possibility to deliver a relatively adequate amount of antigens inside APCs. The other benefit of polymeric carriers is the simultaneous delivery of antigens and adjuvants to APCs to induce the desired immune responses [10,11,12]. As a result, polymeric vaccine delivery systems can bring effective immune responses that are critical for immunity to many pathogens [6].

In this review, we discuss the advances of needle-free vaccine delivery and summarize the several methods of vaccine delivery by chitosan focusing on needle-free immunization.

## 2. Routes of Needle-Free Immunization

### 2.1. Cutaneous Routes

The skin is one of the first choices of tissue for vaccination from the early history of immunization because vaccines delivered through skin route were overwhelmingly successful for smallpox vaccine, bacille Calmette-Guerin (BCG) and yellow fever. Skin is the particularly attractive site of vaccine administration because it consists of a fundamental part of the immune system under it [13]. The skin is the largest immune organ in the human body and it is primarily composed of epidermis and dermis layers. The epidermis is the outermost layer of the skin and it consists of stratified squamous epithelium of 0.1 mm thickness. The primary component of the epidermis is keratinocytes that demarcate the boundary between epidermis and dermis layers. While the keratinocytes are tightly linked to maintaining the strength of the epidermis contributing to its resistance to the passage of foreign particles, these cells also produce a dense lipid that helps form a waterproof barrier of the skin. Besides, keratinocytes produce keratin (a fibrous protein) continuously until they reach the surface of the skin where they are sloughed off to produce the topmost horny layer of the epidermis, known as the stratum corneum. The latter consists of dead keratinocytes of 10–20 μm thickness which is the major obstacle to the administration of vaccine antigen for cutaneous vaccination. The dermis layer, 1.5–3.0 mm of thickness, is enriched with fibroblasts, collagen, elastic fibers and other skin organelles including blood vessels, lymphatic vessels, hair follicles, sweat glands and sebaceous glands. 

Although the exact mechanisms that generate immune responses against the vaccine antigens administered into the skin are not clearly understood, a large number of immature Langerhans cells residing in the epidermis, composing about a quarter of the skin surface area, are believed to play a key role in cutaneous immunization [14]. Upon stimulation by administered antigens through cutaneous route, Langerhans cells function as APCs, capture the antigens by phagocytosis, produce proinflammatory cytokines and migrate to draining lymph nodes [15]. Further, these APCs display major histocompatibility complex molecules and present the antigen to T-helper lymphocytes, a critical step for triggering subsequent immune responses. On the other hand, other immune system components such as dendritic cells, macrophages and T-helper lymphocytes residing or circulating in the epidermis and dermis orchestrate together with Langerhans cells to contribute for the stimulation of adaptive immune responses [16]. This immunocompetence together with its ease of access makes the skin an attractive route of vaccination.

### 2.2. Mucosal Routes

Mucosal routes represent an accessible passageway of entry for many external pathogens into the body. However, these routes are equipped with a mucosal immune system that acts as the first line of defense to protect the body from the foreign invaders. The mucosal immune system is composed of an integrated network of tissues, lymphoid cells and other biomolecules which orchestrate together through a complex cellular processes yielding specific immune responses to eliminate the foreign particles from the body [17,18]. Historically, immunizations through mucosal routes (oral and nasal) were documented several centuries ago in China as a means of vaccine administration against smallpox. But, mucosal immunization received considerable attention only after the advent of polio vaccine that made a prominent impact on the global eradication of polio. Since then, a variety of mucosal routes including oral, nasal, vaginal and rectal have been explored for vaccine delivery but only the oral and nasal routes have so far been commercially used for human vaccines.

#### 2.2.1. Oral Route

The oral route includes the gastrointestinal (GI) tract and hence contains the largest mucosal surface area. GI tract harbors specialized epithelial cells (M cells), located on the follicle-associated epithelium (FAE) in Peyer’s patch of the gut, that uptake the foreign antigens or delivered vaccines and transport them across the epithelial barrier to underlying gut-associated lymphoid tissues (GALT) to initiate immune reactions [5]. GALT is a highly compartmentalized immunological system that consists of various populations of APCs (dendritic cells, T cells) and plasma cells [7]. After transcytosis of antigens in GALT, the antigens are captured by plasma cells to stimulate the production of secretory immunoglobulin A (sIgA), which can bind and eliminate the foreign antigens from the body. On the other hand, the antigens in GALT are undertaken by dendritic cells which further present antigens to T cells to initiate the production of innate and adaptive immune responses.

Although vaccines delivered through oral route produce both mucosal and humoral responses, the efficiency to induce effective immune responses to prevent the diseases depends largely on the vaccine antigens. Hence, only a few oral vaccines (polio, typhoid fever and cholera) are available in the market for human use so far [19]. Not surprisingly, most of these licensed vaccines are based on live attenuated pathogens. The oral route is, however, challenging for the delivery of non-living or sub-unit vaccines, owing to the presence of proteolytic enzymes and acidic environment of the GI tract that degrades the antigens. Several strategies, including the use of biodegradable polymeric particles, have been implemented to protect the antigens in the GI tract [20,21]. In this method, the antigens are encapsulated in polymer microspheres which not only protect the antigens from the harsh environment of GI tract but also enhance the vaccine efficiency through sustained release of antigens. Among the microspheres, PLGA and chitosan microspheres have received considerable attention due to their successful use for the oral immunization of hepatitis B virus (HBV) [22], tetanus toxoid (TT) [23,24] and other antigens [25,26]. Besides, adjuvants are co-administered with antigens in oral immunization to increase the immunogenicity of non-living vaccines. For example, bacterial enterotoxins like cholera toxin (CT) and heat-labile enterotoxin (LT) have been successfully used for the oral immunization of animals [27,28]. However, these enterotoxins are highly toxic for human use [28]. Hence, several mutants and sub-units of CT and LT have been prepared and their efficiency as adjuvants have been evaluated by oral immunization of animals [29]. Consequently, the use of these mutant adjuvants, with no or low residual toxicity, holds a great promise for the future application of vaccines to humans.

#### 2.2.2. Nasal Route

Nasal route serves as another attractive site of vaccine administration as it contains nasopharynx-associated lymphoid tissue (NALT) which is effective at inducing both mucosal and systemic immunity. The intranasal delivery of vaccines stimulates immune responses in the distal mucosal sites including gastric, respiratory and genital tracts [30,31,32]. For example, intranasal delivery of a live attenuated influenza virus vaccine (FluMist), approved by US FDA, is proven to be protective against seasonal infection. Several clinical studies have also confirmed the stimulation of local and systemic immunity by the delivery of nasal vaccines against diphtheria and tetanus [33], hepatitis B [34], whooping cough [35] and *Streptococcus mutans* infection [36]. On the other hand, a variety of live attenuated nasal vaccines, when immunized in rodents, pigs and monkeys, have strongly induced a varying degree of local immune responses confirming the effectiveness of nasal immunization [37]. With the advancement of technology, efforts to develop non-living or sub-unit vaccines for nasal immunization have also continued to advance. Moreover, intranasal immunization requires lower antigen doses, compared to oral immunization, as the antigens do not encounter with acidic and enzymatic barriers of the GI tract.

Nasal vaccines have been formulated in various forms, such as aerosols [38], liposomes [39] and microspheres [40] and delivered through the nostrils together with or without adjuvants [41,42]. The use of adjuvants such as adamantylamide dipeptide [43] and macrophage-activating lipopeptide [44,45] is sometimes obligatory to attain adequately high immune responses with inactivated vaccines. The mutants of CT [46] and LT [47] have also been harnessed as safe adjuvants for nasal vaccine delivery. However, a nasal vaccine of inactivated influenza virus (Nasalflu), introduced in 2000, was soon withdrawn from the market in 2001 due to vaccine-associated complexity possibly originated from LT adjuvant [48]. Recently, a new lipid-based adjuvant (Endocrine) has been demonstrated to be safe and potent for intranasal immunization in both pre-clinical and clinical studies [49,50,51]. Besides adjuvants, another approach to enhance the immune responses of nasal immunization involves the use of chitosan [52]. Studies with the nasal delivery of vaccines using chitosan have achieved the balanced immune responses suggesting that chitosan might have adjuvant or immunomodulatory properties [53]. It is believed that the mucosal immune-stimulating property emerges from the cationic charge of chitosan which is thought to prolong the contact time with negatively charged cell surfaces although the significance of prolonged contact time between vaccine and cells to initiate immune reactions is debatable.

#### 2.2.3. Urinogenital Route

Vaginal or rectal route is a direct approach of immunization for vaccines against sexually transmitted diseases such as human immunodeficiency virus infection and acquired immune deficiency syndrome (HIV/AIDS). However, several factors should be taken into consideration for the development of vaginal vaccines because the vaginal route has some exceptional features in contrast to other mucosal routes. Interestingly, the vaginal mucosa is histologically devoid of organized mucosa-associated lymphoid tissue (MALT) and hence any antigens entered in the vaginal lumen are sampled by macrophages and dendritic cells which then travel towards draining iliac lymph nodes where T-cell priming occurs to switch the migration of T and B cells to the effector sites [54,55]. Moreover, the efficiency of antigen-uptake and the generation of immune responses in the vaginal route are greatly regulated by estrous cycle and sex hormones [56,57,58].

As a proof of concept study, vaginal immunization, using ovalbumin (OVA) with CpG oligodeoxynucleotide adjuvant, has proven to be efficient in priming antigen-specific CD4+ T cells and inducing their transportation from draining lymph nodes to distal lymphoid organs [59]. Similarly, a multi-strain vaccine containing ten heat-killed uropathogenic bacteria, which was efficacious against cystitis in humans when given parenterally, has shown to be efficacious in nonhuman primates when given through the vaginal mucosal route [60]. A recent study has successfully demonstrated the clinical efficacy of vaginal mucosal immunization with a multivalent bacterial vaccine in reducing recurrence of *E. coli* urinary tract infections in women [61]. In another study, vaginal immunization of a novel vaginal ring vaccine device containing recombinant HIV protein plus R848 adjuvant in sheep has elicited robust antigen-specific systemic and mucosal humoral immune responses [62]. Importantly, all sheep displayed mucosal antigen-specific IgA responses 30-fold greater than systemic levels. The first clinical trial of vaginal immunization using only HIVgp140 and HSP70 in women has exhibited to induce a dual innate protective mechanism with significant adaptive CD4+ and CD8+ T cell proliferative responses [63].

Rectal route of immunization, although not common, has been practiced with numerous vaccines. To evaluate whether the rectal route of immunization offers appropriate protection against enteric pathogens, mice were first rectally immunized with rotavirus virus-like particles (VLPs) alone or combined with various toxin adjuvants and finally challenged with rotaviruses [64]. Although the mice immunized with rotavirus VLPs alone showed no protection from rotavirus infection, the mice immunized with rotavirus VLPs combined with some of the toxin adjuvants exhibited complete protection against rotavirus challenge. These outcomes support the possibility to develop new vaccines against enteric pathogens infections if the appropriate adjuvant is given through the rectal route. To determine an optimal route of mucosal immunization to induce a high level of antibodies in the female genital tract and rectum, groups of women were immunized with a cholera vaccine, containing killed *Vibrio cholera* and the recombinant cholera toxin B (CTB) subunit, through oral, vaginal or rectal route [1]. All three immunization routes showed similar levels of antigen-specific immunoglobulin G (IgG) in serum and IgA in saliva. Not surprisingly, rectal immunization was superior to other routes for producing high levels of specific IgG and IgA in rectal secretions while vaginal immunization induced local production of antigen-specific IgG in the genital tract only. Rectal immunization did not generate antibodies in the vagina and vice versa. Thus, the stimulation of optimal immune responses to sexually transmitted organisms in the rectal or the genital route of women appears to require local immunization at the particular site.

## 3. Needle-Free Cutaneous Immunization

Cutaneous methods of immunization can be divided into two major categories, namely injection method and topical application method. While injection methods deliver the vaccines into intradermal, subcutaneous or intramuscular regions, topical application methods deliver the vaccines on or across the skin through facilitated transdermal transport or passive diffusion (Figure 1) [1].

### 3.1. Liquid-Jet Injections

Needle-free liquid jet injection is known as one of the oldest methods for delivering macromolecules in humans. Jet injectors have its origins to the late 1800s, originally used to deliver jets of water and other liquids for different applications. Liquid jet injectors use a high-velocity jet to deliver molecules or vaccines into the intradermal, subcutaneous or intramuscular region of skin. In contrast to needle immunization, liquid jets penetrate a larger tissue volume of skin facilitating the delivered vaccines to contact with a greater number of APCs. As a result, antigens delivered by jet injectors induce similar or higher immune responses than the responses produced by needle immunization [65,66]. Among the jet injectors, multi-use nozzle jet injectors (MUNJIs) garnered much attention from the 1950s to the 1980s because they could deliver vaccine to a large number of people using the same fluid stream and nozzle at a rate of 1000 immunizations per hour. MUNJIs were successful in immunizing humans with vaccines against a large number of diseases, including measles, smallpox, cholera, HBV, influenza and polio. However, an outbreak of HBV infection in the 1980s due to contamination of body fluids through the nozzle of MUNJIs compelled the World Health Organization to discontinue the use of MUNJIs. To eliminate the risk of contamination, the major concern of MUNJIs, disposable-cartridge jet injectors (DCJIs) have been developed which use a new disposable vaccine cartridge and nozzle for each personnel to deliver the vaccine. Compared to MUNJIs, new DCJIs have the capability to use in mass immunization at a rate of 600 immunizations per hour.

### 3.2. Powder Injections

Another specific form of the injector is powder injector which delivers vaccines to the epidermis in a dry powder form and the method is known as epidermal powder immunization (EPI). Although EPI was first demonstrated in the 1980s to deliver deoxyribonucleic acid (DNA) in plants, the method was soon available to deliver both DNA and conventional vaccines to human in the early 1990s [67]. Compared to needle injections, the powder immunization is seemed to be more effective from the preliminary studies on animals. For example, a single immunization of influenza vaccine by EPI in mice elicited a significantly higher serum IgG titer to influenza virus than that induced by intramuscular or subcutaneous injection [67]. When the mice were challenged with the corresponding virus, EPI of influenza vaccine resulted in 100% protection against mortality whereas subcutaneous injection of the vaccine led to 0–75% survival rates. As in other mode of immunizations, commonly used adjuvants can be used to further increase the immune responses to an antigen delivered by EPI. In an animal study, co-delivery of alum and CpG DNA through EPI enhanced the IgG response to diphtheria toxoid (DT) by 25- and 250-fold, respectively [68]. Furthermore, EPI, depending on an adjuvant, selectively activated different subsets of T helper cells and hence different types of immune response indicating that adjuvants in EPI can produce desirable humoral and cellular immune responses.

EPI-based vaccine delivery has been proven to be effective in humans too. A phase I clinical trial conducted for powdered influenza vaccine delivery by EPI elicited high humoral immune responses and seroconversions indicating that EPI is safe in humans [69]. In another study, gold particles-coated DNA vaccine against HBV was delivered by EPI provided the first demonstration of a DNA vaccine stimulating protective antibody titers with both humoral and cellular immune responses in humans [70]. Other clinical studies have also shown the safety and efficacy of a powdered HBV vaccine delivered to the epidermis by EPI [71] and an epidermal DNA vaccine for influenza in humans [72]. These results demonstrate the potential of EPI-mediated vaccine delivery. A major advantage of EPI includes the use of powder vaccine which eliminates the problem for handling and storage. Further studies on the relationship between the physical properties of dry vaccine particles and their mechanism of penetration into the skin would guide in the development of future EPI devices.

### 3.3. Microarray (Microneedle) Patches

**Figure 4:**
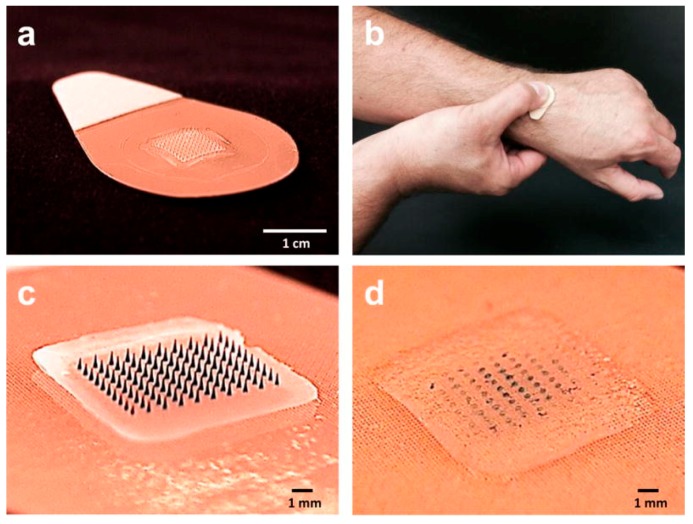
Microneedle patch (MNP) for influenza vaccination. (**a**) The MNP contains an array of 100 microneedles measuring 650 μm tall that is mounted on an adhesive backing. (**b**) The MNP is manually administered to the wrist, enabling self-administration by study subjects. (**c**) Microneedles encapsulate influenza vaccine (represented here by blue dye) within a water-soluble matrix. (**d**) After application to the skin, the microneedles dissolve, thereby depositing vaccine in the skin and leaving behind a patch backing that can be discarded as non-sharps waste. (Reprinted with permission from Ref. [81]).

Microarray patches are sharp microscopic projections constructed into arrays (Figure 4) that can facilitate creating pathways for vaccine entry through the skin [73,74]. The antigens are coated onto the projections of the patches, which when applied on the skin, the antigens gradually penetrate and diffuse into the skin. In an exemplary study, the microarray patches coated with inactivated influenza viruses induced strong immune responses and protected against lethal viral challenge in some murine models [75]. In another approach, Macroflux^®^ microprojection array system, coated with OVA antigen, was evaluated in a hairless guinea pig model. The results demonstrated that antigen delivered by microprojection array system elicited antibody titers, up to 50-fold greater than the same antigen delivered by subcutaneous or intramuscular injection [76]. In their further investigation, they concluded that the immune response produced by microprojection array system is dependent on the dose of the antigens but independent of the depth of delivery, the density of microneedles, or area of application [77]. Importantly, the feasibility of a microneedle patch to deliver live attenuated vaccine without compromising its immunogenicity was demonstrated by a BCG-coated microneedle vaccine patch, which when administered in the guinea pig model, induced a robust cell-mediated immune response in lungs and spleen, comparable to those induced by traditional hypodermic needle-based intradermal BCG vaccination [78]. Other vaccines such as hepatitis C virus DNA vaccine [79] and inactivated rotavirus vaccine have also provided promising results with microneedle patches [80].

Another strategy to deliver antigen through skin used microarray patches with the sharps, made up of biocompatible polymers such as carboxymethylcellulose, which dissolves in the skin with hydration, thus releasing the antigen. To evaluate the dissolving microneedle patches for cutaneous vaccination, three different antigens of influenza virus vaccine strains were delivered individually with the patches in mice [81]. Outstandingly, all three antigens, after a single immunization, induced higher neutralizing antibody titers compared to intramuscular immunization. Besides, the mice immunized with dissolving microneedle patches with influenza vaccine provided complete protection from lethal challenge. They further conducted a clinical trial to compare the safety, immunogenicity and acceptability of the dissolving microneedle patches encapsulating inactivated influenza vaccine [82]. This study showed for the first time in a human clinical trial that influenza vaccination using dissolving microneedle patches was well-tolerated and they induced robust antibody responses.

**Figure 5:**
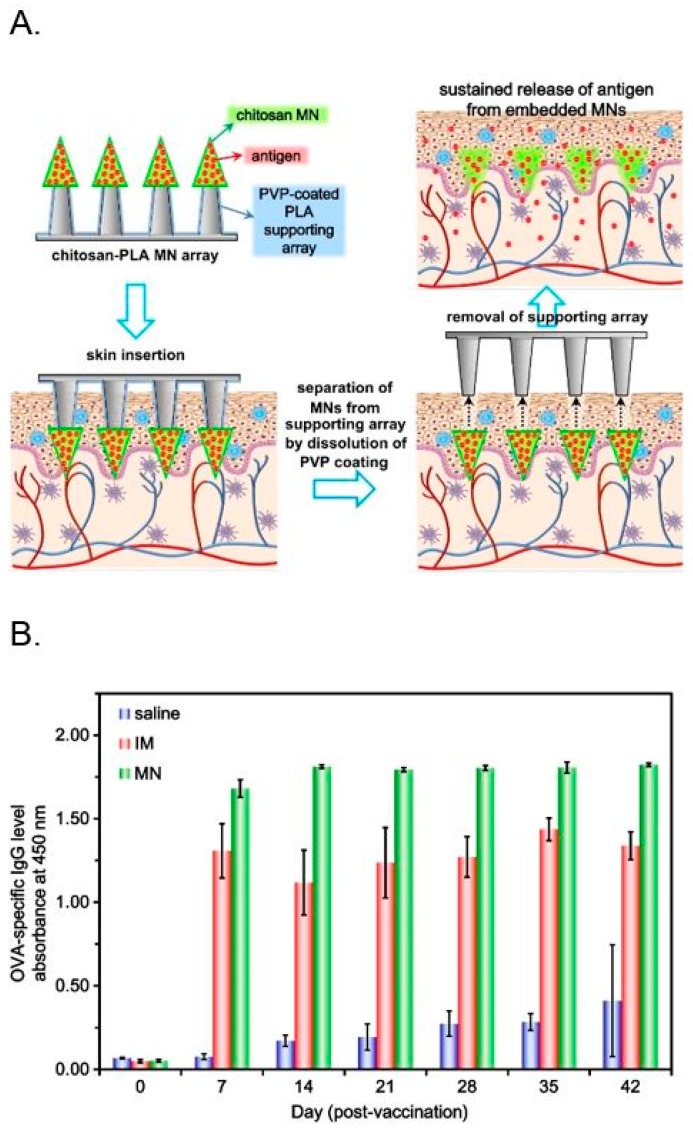
(**A**) Schematic illustrations of sustained transdermal delivery of antigen using a microneedle delivery system, composed of embeddable chitosan microneedles (MNs) and a poly(l-lactide-co-d,l-lactide) (PLA) supporting array. (**B**) OVA-specific IgG levels of rats after a single dose of antigen: non-immunized group (saline), intramuscularly immunized (IM, 1 mg OVA) and microneedle-immunized (MN, 1 mg OVA/array) rats (n = 3 for each group). (Reprinted with permission from Ref. [83]).

A new strategy introduced a microneedle transdermal delivery system consisting of embeddable chitosan microneedles for efficient delivery of encapsulated OVA antigens to the skin (Figure 5) [83]. Chitosan was selected as the microneedle material due to its biocompatible, biodegradable and immune stimulating properties that can induce humoral and cellular responses. The microneedle array was made by mounting OVA antigen-loaded chitosan microneedles on a poly(l-lactide-co-d,l-lactide) (PLA) supporting array to offer mechanical strength to the microneedles during puncture in the skin. After insertion in the skin, the OVA-loaded microneedles separate from the supporting array, slowly releasing the antigen inside the skin and ameliorating antigen presentation. To investigate the effectiveness of the system, OVA-loaded microneedle array system was delivered to rats. When immunized by a single microneedle dose of OVA in rats, the OVA-specific antibody response was significantly higher in the microneedle group than the intramuscular immunization.

In another practical application of microneedle-based vaccination using chitosan, pH-sensitive microneedles were coated via layer-by-layer assembly of inactivated polio vaccine (IPV) and *N*-trimethyl chitosan and subsequent topical application of IPV/chitosan coated microneedles in rats led to the induction of IPV specific antibody responses, demonstrating the efficient delivery of the vaccine [84]. In another attempt to develop microneedle-based vaccination for sustained delivery of antigens, a composite microneedle comprising of a chitosan base and a sodium hyaluronate tip was designed for biphasic release of antigens that would minimize the need for repeated inoculations. Upon application of the OVA-loaded composite microneedles onto skin, the hyaluronate tip dissolves within the skin for immediate release of OVA antigens for priming the immune system, while the biodegradable chitosan base stays in the dermis for prolonged release of antigens, thus further boosting the stimulated immunity [85]. The immunization results showed that a single-dose of OVA with the composite microneedles in rats stimulated considerably higher antibody responses than a traditional two-dose of subcutaneous vaccination.

## 4. Needle-Free Immunization with Chitosan Systems

Chitosan, a linear amino polysaccharide consisting of β1,4-linked monomers of d-glucosamine and *N*-acetyl-d-glucosamine, is obtained by partial deacetylation of chitin, a long-chain polymer of *N*-acetylglucosamine. Hence the degree of deacetylation directly affect the physical properties (molecular weight, solubility and biodegradability) and biological properties (cytotoxicity, antigen binding capacity and adjuvant activity) of chitosan [86,87,88]. Because chitosan is insoluble in water at neutral pH, many soluble chitosan derivatives have been produced by substituting amine and/or hydroxyl functional groups of chitosan with hydrophilic groups such as thiol [89,90], sulphate [91] hydroxyalkyl [92,93], carboxyalkyl [94] and succinyl [95]. Other methods involve the use of poly(ethylene glycol) (PEG) [96] and Poloxamer [97] to enhance the solubility of chitosan. Among the derivatives, quaternized *N*-alkyl chitosan derivatives behave as cationic polyelectrolytes which are highly soluble in water [98]. They also have mucoadhesive and penetration-enhancing properties which, however, depend on the degree of substitution or quaternization in chitosan.

Apparently, the mucoadhesive property of chitosan in mucosal route emerges from the electrostatic interaction between the cationic charge of chitosan and negatively charged mucins. Thus, chitosan particles can reside longer in the mucosal surfaces leading to a higher possibility of particles to enter through M cells and/or epithelial cells in the mucosal routes [52]. Due to these consequences, chitosan derivatives have been developed as antigen delivery carriers which are supposed to assist the transportation of antigens to mucosal immune compartments, prolong the interaction time between antigen and immune cells and initiate the immune reactions from local to distal sites. The other advantage of chitosan carrier systems is the ease of tuning the release of antigens in the mucosal sites because different physicochemical properties such as solubility, surface charge and hydrodynamic size of the chitosan particles can vary the release pattern of antigens from the chitosan particulate systems. Hence, the delivery of antigens in polymeric particulates, either as microparticles or nanoparticles, has been employed as a successful approach for mucosal delivery [99,100].

### 4.1. Formulations of Chitosan Particles

Chitosan-based particulates for needle-free vaccine delivery can be formulated by ionic interaction between cationic chitosan derivatives and anionic crosslinking substrates such as tripolyphosphate (TPP) or sodium sulfate. The spontaneous self-assembly of oppositely charged particles gives rise to microparticles or nanoparticles depending upon the molecular weights of chitosan derivatives and crosslinkers. Usually, protein-loaded chitosan particulates have been formed by ionotropic gelation of chitosan with TPP. In this process, an aqueous solution of antigens with TPP is stirred with an aqueous solution of chitosan to form nanoparticles at room temperature. The process, therefore, avoids organic solvents and high temperature and hence protects the protein or antigen integrity [101,102,103]. For example, chitosan nanoparticles loaded with TT through ionotropic gelation method was investigated as vaccine delivery vehicles through nasal route [104]. Similarly, chitosan nanoparticles were developed as a carrier system for nasal delivery of an influenza subunit vaccine by stirring a solution containing influenza A subunit H3N2 with TPP in a solution of *N*-trimethyl chitosan (pH 7.4) at room temperature. Intranasal immunization with influenza subunit vaccine-loaded chitosan nanoparticles induced strong IgG and IgA levels [105]. Alternatively, ionic crosslinking of sodium sulfate with an aqueous solution of chitosan could produce chitosan particulates with porous structures [106]. As a result, a large number of antigens could be richly loaded by entrapping within the porous particles or protruding on the outer surfaces of chitosan particulates. 

Another promising formulation of needle-free vaccine delivery involves the use of antigen conjugates with chitosan derivatives. The chemical crosslinking of trimethyl chitosan with OVA antigens has ameliorated the immunogenicity of the antigen [107]. Besides, nasal immunization of trimethyl chitosan-ovalbumin conjugates has generated high levels of secretory IgA in nasal washes and higher titers of OVA-specific IgG in mice [108]. However, the aggregation behavior of chitosan particles has hindered their efficient use in vaccine delivery. To minimize the aggregation problems, either chitosan or antigen can be conjugated with PEG that offers improved stability and water solubility to conjugated substances. In a typical example, chitosan was covalently conjugated to PEG, stirred with TTP to prepare stable microspheres and further loaded with *Bordetella bronchiseptica* dermonecrotoxin (BBD) antigens to develop an effective nasal vaccine against atrophic rhinitis in animals [97]. Thus, BBD-loaded pegylated chitosan microspheres, owing to their efficient release of antigens and subsequent stimulation of cytokines from macrophage cells in vitro, has represented as a promising vaccine delivery system for nasal immunization.

Apart from PEG, Poloxamer or Pluronic has been utilized in many vaccine formulations for needle-free immunizations. In an attempt of mucosal delivery of TT antigen, a mixture of chitosan and Pluronic F-127 boosted through nasal route, following the first immunization of TT antigen through intraperitoneal injection, showed a significant improvement in the systemic antigen-specific IgG response and mucosal IgA response in the nasal and lung washes in mice [109]. The results indicated that Pluronic may act as an adjuvant to induce an additive or synergistic effect on mucosal immunization of vaccines.

In another study, chitosan microspheres, prepared with BBD antigens in the presence of Pluronic F-127, showed a higher amount of antigen release and stimulated a higher level of immune responses in mouse alveolar macrophage cells in vitro [96]. Further, nasal immunization of BBD-loaded Pluronic F-127/chitosan microspheres induced higher BBD-specific IgA responses in nasal secretions in the mice. Moreover, these immunized mice, when challenged with *B. bronchiseptica* through nasal route, survived longer than the control groups, demonstrating the potential of chitosan particulates for nasal delivery of vaccines.

### 4.2. Mucoadhesive Chitosan Particles

Mucoadhesive chitosan microspheres can be simply produced by coating chitosan microspheres with a mucoadhesive polymer to enhance the stability and efficiency of microspheres for mucosal delivery of vaccines. Accordingly, a mucoadhesive and pH-sensitive polymer, Eudragit, was coated on chitosan microparticles to deliver OVA antigen in mice [110]. Oral immunization of OVA by Eudragit-coated chitosan microparticles induced high levels of OVA-specific IgA and IgG in fecal and plasma, respectively. Similarly, the coating of thiolated Eudragit on chitosan microspheres containing bovine serum albumin (BSA) antigen not only protected the release of BSA from the microspheres at gastric pH but also increased the mucoadhesive potential of the microspheres in mucosal tissues in vitro and in vivo [111]. In the same way, another pH-sensitive polymer, hydroxypropyl methylcellulose phthalate (HPMCP), was mixed with trimethyl chitosan in the presence of hepatitis B surface antigen (HBsAg) to form nanoparticles by self-assembly [112]. Further experiments showed that HPMCP protected the release of HBsAg from chitosan nanoparticles at gastric pH, suggesting that HPMCP-coated chitosan nanoparticles could be harnessed for HBsAg delivery by the oral route. In another study, the efficacy of glycol chitosan as a mucoadhesive coating material to deliver HBsAg through nasal route was investigated [113]. Owing to higher mucoadhesive potential, glycol chitosan nanoparticles showed low nasal clearance thus increasing the mucosal uptake of antigen which eventually enhanced humoral and mucosal immunity. To demonstrate the effect of mucoadhesive coating on the delivery carriers for mucosal vaccines, glycol chitosan was coated on HBsAg-loaded PLGA nanoparticles and delivered through nasal route [114]. The results showed that the lower nasal clearance of glycol chitosan coated-PLGA nanoparticles, compared to uncoated PLGA nanoparticles, resulted in higher uptake of antigens through local or systemic circulation, to produce higher mucosal and systemic immune responses.

### 4.3. Targeted Chitosan Particles

**Figure 6:**
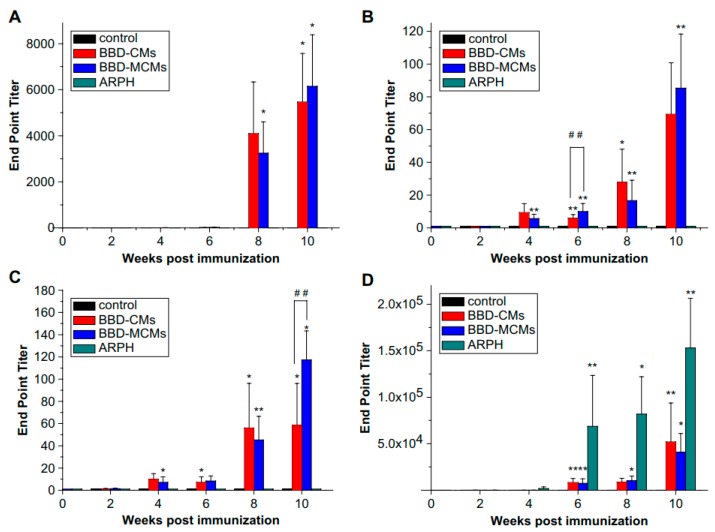
Anti-BBD IgA levels in (**A**) nasal wash; (**B**) saliva; (**C**) serum and (**D**) anti-BBD IgG levels in serum (data are means ± standard deviations, n = 3). Significant differences between untreated and immunized groups was expressed as * *p* < 0.001 and ** *p* < 0.05 and between BBD-CMs and BBD-MCMs groups as ^##^ *p* < 0.05. (Reprinted with permission from Ref. [116]).

Undoubtedly, chitosan particles are promising carriers for needle-free vaccine delivery. However, the antigen delivery or targeting efficiency of chitosan particles varies with the particular cells or tissues. This issue can be solved by selective targeting of cells through ligand-receptor interaction. Thus, chitosan particles or antigens can be conjugated with various ligands or antibodies which can specifically bind to the specific cells or their receptors. APCs and M cells have been selected as two common targeted cells for vaccine delivery by chitosan particles. Because mannose receptors are surrounded on the surfaces of APCs such as macrophages [115], mannose as a specific ligand was conjugated with chitosan microspheres and used them for nasal delivery of vaccines [116]. Accordingly, mannosylated chitosan microspheres (MCMs), loaded with BBD antigens, exhibited high binding affinity with macrophage cells. Nasal immunization of BBD antigen with MCMs showed higher antigen-specific IgA responses in saliva and serum of mice than nasal immunization of BBD antigen with chitosan microspheres only (Figure 6). The results concluded that the high level of immune responses, although partly contributed by adjuvant property of chitosan, was majorly due to higher uptake of MCMs in macrophage cells through ligand-receptor interaction.

As stated earlier, conjugation of receptor-specific antibodies to antigens is an alternative approach of targeting APCs for selective delivery of antigens [117,118]. It is now well-documented that the differences in levels of cellular and humoral immune responses primarily depend upon the type of interactions between antibodies and receptors on APCs [119,120]. Therefore, many studies have focused to find novel ligands which can strongly bind to the receptors of APCs to stimulate appropriate immune responses. In an attempt to find an effective ligand for dendritic cells, a novel peptide, TPAFRYS (TP) was isolated by phage display technique. Elegantly, TP-conjugated chitosan nanoparticles had stronger binding specificity towards dendritic cells than myoblasts or macrophages [121]. Further, subcutaneous immunization of TP-conjugated chitosan nanoparticles with OVA antigen exhibited high production of OVA-specific serum IgG in mice demonstrating the efficacy of targeted delivery of vaccines in APCs.

Because M cells play a key role as the entry point for particles or antigens into lymphoid tissues to induce immune reactions of mucosal vaccination [7], various M cell targeting ligands have been entertained for mucosal vaccines [122]. Lectins are one of the natural ligands that have high affinity with sugar moieties on intestinal cells [123]. For example, Ulex europaeus agglutinin I (UEA1), an α-l-fucose-specific lectin, owing to its specificity with M cells has been frequently linked with delivery carriers of mucosal vaccines [124]. Hence, the efficacy of UEA-1 to target murine M cells and subsequent induction of immune reactions was evaluated by comparing the efficiency of antibodies production between oral immunization of UEA-1-coated microparticles with HBsAg and intramuscular immunization of alum with HBsAg [125]. While both routes of immunization produced similar levels of HBsAg-specific antibodies, oral immunization also elicited sIgA responses in vaginal, intestinal and salivary secretions [125]. In the further comparative study, BSA-loaded chitosan nanoparticles, coated with UEA-1-alginate, were administered through oral route while BSA-loaded chitosan nanoparticles with alum were immunized in mice through parenteral route [126]. The results revealed that oral immunization of antigen by UEA1-alginate-coated chitosan nanoparticles elicited superior systemic response plus dominating mucosal response than the antigen delivered by parenteral immunization. These data show the potential of chitosan nanoparticles combined with targeting ligand as an effective delivery system for oral immunization.

Some peptides that have specific binding capacity with M cells have been identified and exploited for M cell targeting vaccine delivery. These peptides are usually discovered by both in vitro and in vivo phage display screening technology. For example, VPPHPMTYSCQY (P25) and LETTCASLCYPS (P8) peptides, identified by in vivo screening method, have shown strong binding on the surfaces of intestinal tissues while another peptide, YQCSYTMPHPPV has demonstrated to assist the efficient delivery of polystyrene particles to M cells in a mouse model [127]. In another study, CTGKSC and LRVC peptides, identified by in vitro phage display method, have enhanced the transportation of polycaprolactone-PEG nanoparticles into the FAE through M cells in the intestine [128]. Another peptide, SFHQLPARSPLP (Co1), selected by screening phage display library, was fused with enhanced green fluorescent protein (EGFP) antigen to determine the ability of Co1 to deliver antigen by targeting M cells [129]. Compared to EGFP alone, the fused EGFP-Co1 showed higher binding affinity to M cells, entered easily in Peyer’s patches and subsequently elevated the production of fecal IgA and serum IgG, signifying Co1 as a promising ligand for M cell-targeted antigen delivery.

**Figure 7:**
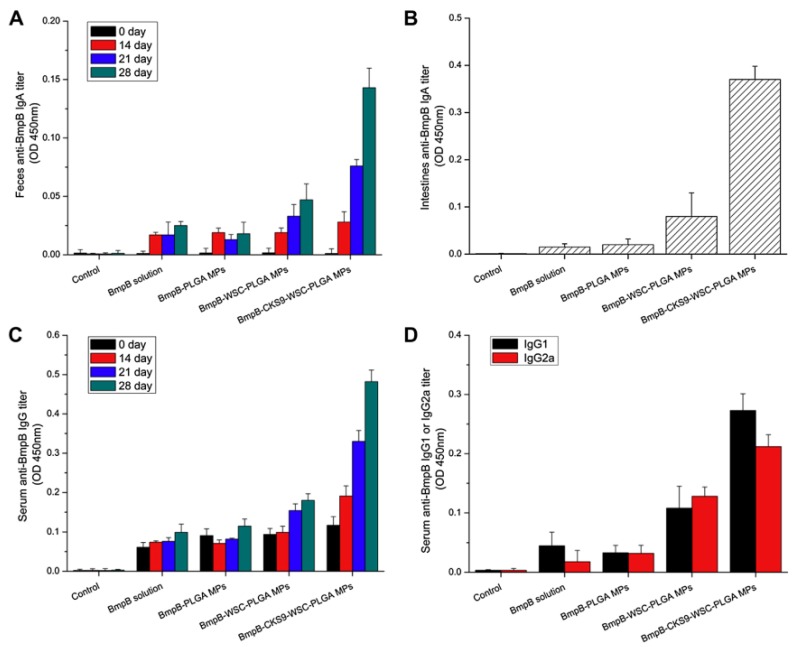
BmpB-specific immune response detection by ELISA after oral administration. Anti-BmpB IgA levels in feces (**A**) and intestine (**B**), anti-BmpB IgG levels in serum (**C**) and anti-BmpB IgG1 and IgG2a (**D**) levels. (Reprinted with permission from Ref. [131]).

Similarly, an M cell-homing peptide, CKSTHPLSC (CKS9), selected by the phage display technique, was chemically conjugated to chitosan nanoparticles (CKS9-CNs) and determined the targeting ability of CKS9-CNs to M cells [130]. Compared to chitosan nanoparticles, CKS9-CNs were significantly accumulated into Peyer’s patches in small intestines of rat. These results indicate that CKS9, owing to its enhanced targeting and transcytosis ability of particles, could be used for efficient delivery of mucosal vaccines. Accordingly, a vaccine was developed by loading a model antigen, *Brachyspira hyodysenteriae* membrane protein B (BmpB) into PLGA microparticles, which were further coated with CKS9-conjugated chitosan and administered in mice through oral route [131]. Oral immunization of CKS9-chitosan-PLGA microparticles with BmpB vaccine exhibited enhanced levels of IgG in serum and sIgA in both intestinal and fecal secretions (Figure 7). These data indicated that the use of M cell targeting peptide could improve the effectiveness of oral immunization of particulate vaccines.

### 4.4. Adjuvant Activity of Chitosan Particles

An immunologic adjuvant is defined as any substance that tends to accelerate, enhance or prolong antigen-specific immune responses of vaccine antigens [132]. The intrinsic activity of polymers to stimulate various cellular functions of cells and immune cells gives rise to the adjuvant property to polymers [133]. Numerous studies have evaluated the potential of biodegradable polymers as adjuvants including PLGA and chitosan. Chitosan and its derivatives have been studied in numerous vaccine formulations and shown the adjuvant activity when given in combination with the vaccines [134]. Chitosan-based intranasal vaccine against hepatitis B was formulated to study the adjuvant effect of the chitosan and its underlying mechanism of action. In vivo and in vitro experiments exhibited increased residence time of HBsAg encapsulated chitosan particles in the nasal cavity due to the interaction of positively charged particles and the negatively charged mucosa of the nasal cavity. In addition, the decrease in transepithelial electrical resistance (TEER) values with a consequently increased transport of HBsAg across the monolayered human colonic carcinoma-derived Caco-2 cells was observed in the presence of chitosan but not in the group without chitosan, indicating that chitosan is capable of opening tight junctions. It further showed that chitosan increased the uptake of antigens by dendritic cells and promoted their maturation. Mice immunized with HBsAg plus chitosan showed the significantly increased level of anti-HBs sIgA, IFN-γ- and IL-2 than the mice immunized with alum-based vaccine and plain HBsAg [135].

Another study evaluated the effects of alginate modification on absorption properties of fluorescein isothiocyanate (FITC)-BSA loaded *N*-trimethyl chitosan chloride (TMC) nanoparticles. In addition, the feasibility of applying TMC nanoparticles loaded with a model vaccine urease, a vaccine protein against *Helicobacter pylori* infection, in oral vaccination was studied. TMC nanoparticles modified with alginate showed higher FITC-BSA permeation efficiency as compared to non-modified TMC nanoparticles. Further, in vivo studies showed that orally administrated urease loaded TMC nanoparticles were able to induce a significantly higher titers of both IgG and IgA as compared with those mice who were immunized with either urease solution or urease co-administered with TMC solution, indicating that TMC nanoparticles are potential carriers for oral protein as well as vaccine delivery [136].

Recently, a study reported that intranasal administration of chitosan alone could completely protect BALB/c mice from lethal infection by H7N9 virus, a highly pathogenic virus, by the stimulation of the innate immune system. In vivo experiments showed that mice challenged with lethal dose of H7N9 (10 × LD_50_) could be protected even after ten days of the intranasal chitosan administration. It further demonstrated that the infiltration of leukocytes in the bronchoalveolar lavage and proinflammatory cytokines were significantly enhanced in the lungs of mice treated with chitosan as compared with untreated groups, indicating the potent activation of mucosal immune responses by intranasally delivered chitosan [137].

Similarly, chitosan has exhibited to induce various cytokines, interleukin (IL)-1 and colony-stimulating factor (CSF) in macrophages in vitro [138]. Moreover, chitosan derivatives have shown their adjuvant activities such as activation of peritoneal macrophages, suppression of tumor growth and protection of the host against bacterial infection in mice and guinea-pigs [87,88]. Similarly, chitosan has significantly elevated the number of T cells, dendritic cells and natural killer cells in herpes simplex virus (HSV) infected mice [139]. Further, the adjuvant function of chitosan was compared with a standard vaccine adjuvant, cholera toxin (CT), in a study of vaccine delivery in mice infected with *Helicobacter pylori* [140]. Oral immunization of the vaccine with chitosan had higher or comparable humoral immune response, Th1/Th2 cell immune reaction and *H. pylori* elimination rate than the same vaccine administered with CT. In another study, mice were challenged with a lethal dose of various influenza viruses following the nasal immunization of a vaccine containing a matrix protein 1 in combination with chitosan to mice [141]. Nasal immunization with chitosan effectively protected the mice against the challenge of both homologous and heterologous influenza viruses to various extents.

An informative study was designed to gain insight into the immunogenicity of *N*-trimethyl chitosan, either in solution or nanoparticle formulation, by delivering diphtheria toxoid (DT) through transcutaneous route in combination with microneedles [142]. Microneedle-based transcutaneous immunization of DT with or without chitosan solution formulation elicited 8-fold higher IgG titers in the latter than the former. However, neither topically applied DT-loaded chitosan nanoparticles were able to enhance the IgG titers compared to DT alone nor microneedle application could improve the immunogenicity of the DT-loaded chitosan nanoparticles. The consequence was due to the limited transport of the chitosan nanoparticles into the skin using the microneedle conduits compared to chitosan solution. It means the combination of microneedles and adjuvant can assist the transport of antigens into the skin and the activation of the APCs for successful transcutaneous immunization. The study revealed that chitosan in solution offers great potential as an adjuvant for transcutaneous immunization with microneedles but not when formulated in nanoparticles.

To enhance immunogenicity of antigens with low-dose immunization through sustained intradermal delivery, a system comprising OVA-loaded chitosan microneedles with dissolving patches was developed and tested in rats. The immunization results showed that rats administered with chitosan microneedles containing low-dose OVA (200 μg) had significantly higher antibody levels than those administered with intramuscular injection of full-dose OVA (500 μg) [143]. Moreover, the delivery of OVA with chitosan microneedles induced significantly stronger immune responses compared with the delivery of same dose of OVA and chitosan by intramuscular injection indicating chitosan microneedles as an efficient vaccine delivery system with enhancing adjuvant activity.

## 5. Conclusions and Prospectives

Chitosan-based systems have demonstrated as one of the most promising delivery vehicles for needle-free immunizations. A major advantage of biodegradable and biocompatible chitosan is that it also acts as adjuvant for the delivered antigens. Besides, chitosan can be conjugated with ligands to develop carriers for targeted delivery. Moreover, chitosan can be produced with various formulations (solid, liquid or gel) with a diverse range of particle sizes. As a delivery carrier, chitosan can shield antigens from degradation, enhance the residence time of antigens at mucosal surfaces and introduce antigens to the immune compartments for effective induction of both mucosal and systemic immunity [144]. Despite several advantages, the mechanism of immune reactions by chitosan is still not well understood. Since the induction of immune responses is dependent on the physicochemical properties of both antigens and chitosan particles, more studies at molecular levels are required to optimize chitosan carriers for effective needle-free immunizations.
